# Economic Benefits of Waste Pickling Solution Valorization

**DOI:** 10.3390/membranes12020114

**Published:** 2022-01-19

**Authors:** Rosa Gueccia, David Bogle, Serena Randazzo, Alessandro Tamburini, Andrea Cipollina, Daniel Winter, Joachim Koschikowski, Giorgio Micale

**Affiliations:** 1 Dipartimento di Ingegneria, Università Degli Studi di Palermo, 90128 Palermo, Italy; rosa.gueccia@unipa.i (R.G.); serena.randazzo@unipa.it (S.R.); alessandro.tamburini@unipa.it (A.T.); giorgiod.maria.micale@unipa.it (G.M.); 2 UCL Department of Chemical Engineering, London WC1E 6BT, UK; d.bogle@ucl.ac.uk; 3 Fraunhofer Institute for Solar Energy Systems ISE, 79110 Freiburg, Germany; daniel.winter@ise.fraunhofer.de (D.W.); joachim.koschikowski@ise.fraunhofer.de (J.K.)

**Keywords:** industrial wastewater, pickling, economic analysis, optimization, diffusion dialysis, membrane distillation

## Abstract

An integrated hybrid membrane process, composed of a diffusion dialysis (DD), a membrane distillation (MD) and a reactive precipitation unit (CSTR), is proposed as a promising solution for the valorization and onsite recycling of pickling waste streams. An economic analysis was performed aiming to demonstrate the feasibility of the developed process with a NPV of about EUR 40,000 and a DPBP of 4 years. The investment and operating costs, as well as the avoided costs and the benefits for the company operating the plant, were analyzed with an extensive cost tracking exercise and through face-to-face contact with manufacturers and sector leaders. A mathematical model was implemented using the gPROMS modelling platform. It is able to simulate steady state operations and run optimization analysis of the process performance. The impact of key operating and design parameters, such as the set-point bath concentration and the DD and MD membrane areas, respectively, was investigated and the optimal arrangement was identified. Finally, operating variables and design parameters were optimized simultaneously in a nonlinear framework as a tradeoff between profitability and environmental impact. We show how the integration of new technologies into the traditional pickling industry could provide a significant benefit for the issues of process sustainability, which are currently pressing.

## 1. Introduction

The harsh and outdated hot dip galvanizing practice is still the primary process used globally for metal surface treatment, along with the pretreatment pickling process for scale and impurities removal from steel products. Pickling waste liquors pose a crucial problem for company economics and, even more, for its consequent environmental impact. AIZ (The Italian Galvanizing Association) carried out a survey within the framework of the ReWaCEM European project (www.rewacem.eu, accessed on 8 December 2021), thus obtaining information on acid consumption and spent liquor production data used in pickling in Italy. A fresh acid consumption range of 10–30 kg/ton_galv.steel_ and a spent liquor production of 15–45 kg/ton_galv.steel_ are reported [1]. These data are consistent all over Europe according to surveys conducted by the EGGA, the European General Galvanizers Association community.

The oldest and most straightforward way to deal with spent pickling solution is the neutralization and subsequent disposal method which is no longer considered a Best Available Technique (BAT) [2]. The IPPC (Integrated Pollution Prevention and Control) policy pushes for the adoption of innovative integrated and eco-friendly systems for recovery of acids and metal compounds by applying the circular economy concept. This has the simultaneous goal of (i) reducing the environmental impact and (ii) making the production steps more efficient.

With this increasing attention to environmental aspects alongside profitability, an integrated hybrid membrane process composed by a diffusion dialysis (DD), a membrane distillation (MD) and a reactive precipitation unit (CSTR) is presented here. The process feasibility was studied and proposed in previous authors’ works. Process design was carried out by adopting an earlier developed process simulator [3] and relevant experimental evidence was demonstrated both at laboratory and pilot scale [3,4,5,6].

Here, economic considerations regarding the integration of the process in the industrial environment are presented, firstly for a hot-dip galvanizing company in Italy (Tecnozinco S.r.l., Carini, Italy, case study) and then also considering medium-size throughput treatment plants. Diffusion Dialysis is counted in the BAT recommendations [7], as it has widely proved its clean nature [8,9]. Despite the evidence reported in the literature in cost effectiveness, where it is only marginally attractive [10,11], issues related to the stand-alone application (e.g., large volume of product streams [11]) can be overcome using an integrated approach. More promising results are expected for the MD application, especially when it can be sustained with waste heat [12], as occurs in our case.

The huge potential of considering an onsite treatment process in the galvanizing industry concerns (i) the reduction or total elimination of the disposal waste to treat and (ii) the avoidance its transportation towards treatment sites. The present work is devoted to exploring such potential and in particular to proving that the integration of the treatment and recycling process will be a cost-effective solution, as already found for different recovery processes in the same field [13]. The integration of the process in the industrial plant does not require any particular modifications of the site. Moreover, the flexibility and size-modularity of membrane units enables the size to be adapted together by adjusting operating conditions to any specific galvanizing plant needs.

Introducing further environmental aggravations should be avoided, as could happen by increasing the process water consumption with related increasing water streams flow rate to be included in the industrial water circuit [14]. An optimization analysis is required to create more insight in the proposed process and to increase the attraction for the relevant industry. Several configurations were explored by varying design and operating variables to determine the most profitable configuration. A multi-objective problem formulation approach was followed in order to consider also freshwater consumption alongside the economic point of view [15].

## 2. Process Modelling Platform

**Figure 1:**
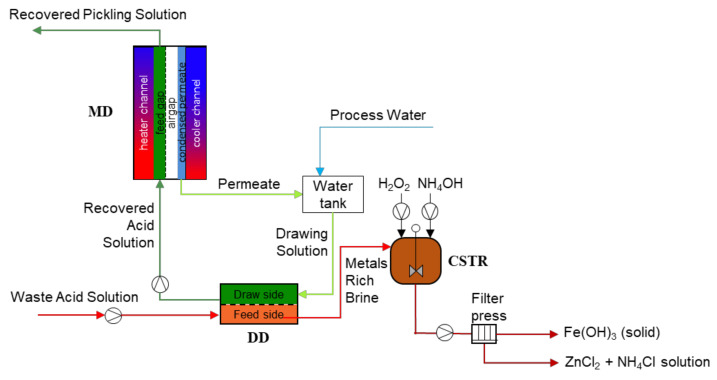
Schematic representation of the integrated hybrid membrane process.

In Figure 1, a schematic representation of the integrated system is shown. This process representation is adapted from the process flow diagram published in the authors’ previous work [6]. The waste acid solution is extracted from the pickling bath and sent to the Diffusion Dialysis (DD) unit. The core element of DD unit is an anion exchange membrane (AEM), characterized by a high permeability of anions and high rejection of main cations. However, due to the very small size and large mobility of protons (H^+^), these can easily pass though the AEM with the chlorides, thus allowing the recovery of the free acid thanks to the diffusive mass transport driven by the concentration difference between the waste acid and the recovered acid solutions. A detailed description of the process was provided in a previous authors’ paper [5].

The recovered acid solution, still at a relatively low acid concentration, due to the need of keeping a large driving force in the DD unit, is processed in a Membrane Distillation (MD) unit. In MD, an intrinsically hydrophobic microporous membrane separates the heated acid solution from a cold channel. Driven by the vapor pressure difference (induced by the temperature difference at the two sides of the microporous membrane) water vapor (generated by the liquid–vapor equilibria at the hot liquid–membrane interface) passes through the membrane pores and condenses on the other side of the membrane on a condensing surface kept cold by a an external cooling water stream. Such condensed low concentration acid solution is, eventually, used as DD drawing solution after the mixing with another freshwater stream. On the other side, the solution flowing in the MD hot channel is concentrated in hydrochloric acid and less volatile components, such as the metal salts, and is recycled back to the pickling bath. The MD heating requirements are accomplished by exploiting the available industrial waste heat through the integration of the technology with the industrial heating water network. More details of the process were provided in a previous paper by the authors [3].

The brine rich in metals exiting from the DD process is directed to a reactive crystallizer (CSTR), where iron (II) ions are oxidized into iron (III) ions by the addition of hydrogen peroxide and precipitated by addition of ammonia, while zinc chlorides and ammonium chlorides remain in solution, thanks to their high solubility, thus generating the so-called fluxing solution, useful in a step of the hot-dip galvanizing chain. Of interest, a detailed description of the crystallization process was provided in a previous authors’ paper [16].

A pilot scale plant of the proposed process was recently designed, built and installed at the Tecnozinco S.r.l. hot-dip galvanizing company in Carini (Sicily, Italy). Detailed characteristics of the process and the pilot system are reported in the authors’ previous work [6].

An integrated model of the entire treatment chain was already developed and implemented using an Excel spreadsheet in order to simulate the stationary operation of the process [3]. In the present work, the model was readapted in order to include plant scaling equations and implemented in gPROMS Model Builder*^®^* simulation platform. All the main equations of the integrated model for the different sections, consisting in simple mass balance and transport equations, are summarized in Table 1. The model was integrated with the economic analysis section described in Section 3, where the main equations are reported too.

## 3. Engineering Economic Analysis

A standard profitability analysis was carried out by following a well-known procedure proposed by Turton et al. [18] to evaluate the economic profitability due to the introduction of the proposed innovative integrated process in the hot-dip galvanizing industry. Compared to the very preliminary and rough economic analysis for the pilot system previously elaborated [3], the present study shows a thorough economic analysis for the full integration of the process in the industrial environment. Moreover, data were significantly refined in order to provide more reliable results and guarantee the following optimization at large scale to be worthy for industry.

Pilot plant capacity was designed in order to be able to treat only one of the seven pickling baths of the Tecnozinco S.r.l. plant. Here, for a consistent (and more valuable for industry) economic analysis, the capacity of the integrated treatment process was scaled-up in the calculation to the full Tecnozinco capacity (2030 kg/h of processed steel, corresponding to 0.13 m^3^/h of treated acid). As Tecnozinco S.r.l. can be considered a small company in the hot-dip galvanizing sector, higher capacity plants were investigated in order to consider the case of a more significant economical return as expected for a larger portion of companies.

### 3.1. Capital Investment

The Fixed Capital Investment (FCI) estimation was assessed through an extensive cost tracking carried out during the realization of the pilot unit and with the help of manufacturers. Results of the analysis for the demo plant and for the up-scaled sizes are reported in Table 2. The “scale variable” is the waste acid volume flow rate (*F^WAS^*) to be treated in the integrated process, as it represents the capacity variable of the proposed treatment process. Costs reported in Table 2 are the sum of costs for parts and modules, which account for the Total Material Costs, and transportation, logistics, documentations, assembly, commissioning and training and development. As shown in Table 2, the cost of some categories was estimated based on the number of person-months (PM) required, by considering the relevant salaries reported in Table 3. Typical Italian engineer salaries are reported [19], which are quite similar for other European countries. The technology providers fee was assumed to be 25% of the subtotal. The sum of all these cost items provides the FCI. Notably, being a waste treatment process to be integrated in an already existing plant, the costs for auxiliary facilities’ development (i.e.**,** green field development costs or grass root costs [20]) are not included. The land cost was assumed to be zero, as the treatment process footprint is small compared to the industrial plant one. As reported in Table 2, the FCI needed to build a “pilot size” treatment process is about EUR 140,000. This value should be regarded as the investment need to install a pilot scale treatment process for commercial use. Note that the FCI at this pilot scale is reported here only because it represents a reliable starting point on which the FCI analysis (i.e., FCI as a function of treatment capacity/scale in terms of treated waste acid) is based. However, the pilot scale will no longer be considered for economic and optimization considerations in the following work and the economic analysis will be referred to the processed manufactured steel (expressed in kg/h).

A *specific membrane module cost* was derived from data provided by DD and MD module manufactures and values of EUR 470/m^2^ and EUR 310m^2^ for the DD and the MD modules were obtained, respectively. In particular, these costs refer to the whole technology and not only to the membrane cost. The FCI can be conceived as the product of a “lump factor” and the cost of the membrane technologies (Equation (34)). The lump factors at different scales were extrapolated from the corresponding FCI values, thus resulting in a power law of the treatment capacity (Equation (35)). This equation will be used later on in the optimization section (Section 4).

(34) $$FCI=lump\;factor\cdot \underset{u=DD,MD}{\sum }\left(specific\;membrane\;module\;cost_{u}\cdot A_{u}\right)$$

(35) $$lump\;factor=4\cdot F^{WAS}{}^{-0.225}$$

where *A_u_* is the total membrane area of either DD or MD modules.

**Figure 2:**
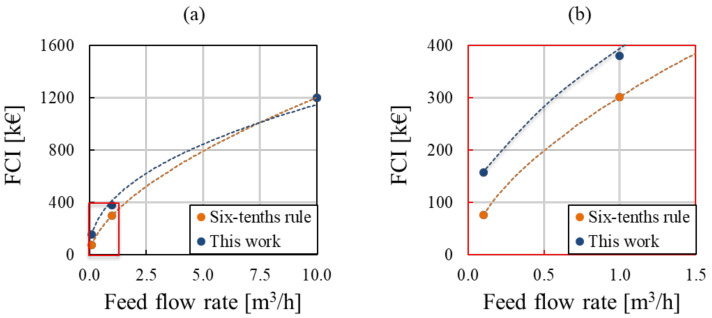
Trend of Fixed Capital Investment versus feed flow rate (plant capacity) obtained in the present work (blue curve) and by the six-tenths rule (orange curve) for a capacity ranging between (**a**) 0.1–10 m^3^/h and (**b**) 0.1–1.5 m^3^/h (in fact, (**b**) is a zoom of (**a**)).

For a validation of the trend obtained for the Fixed Capital Investment vs. the plant capacity (feed flow rate), a comparison with the well-known “*six tenths rule*” was performed (see Figure 2). The “*six tenths rule*” can be expressed as follows [20]:

(36) $$C_{a}=C_{b}\;{\left(\frac{N_{a}}{N_{b}}\right)}^{0.6}$$

where *C* is the purchase cost of the equipment and *N* represents an “attribute” of the equipment itself (e.g.**,** tank volume, treatment capacity, feed flow rate, etc.). The subscript *a* indicates the equipment with the required “attribute”, while *b* indicates the equipment with the base attribute, at which normally the purchase cost is known. In the present case, the feed flow rate can be chosen as “attribute” and the law can be plotted for comparison with the values reported in Table 2.

The curve obtained in the present work is more conservative and predicts higher investment cost for the low- and medium-size capacity plants. The results obtained are comparable within a wide range of capacity, estimated (1 m^3^/h–10 m^3^/h), while a higher discrepancy is observed in the low-size range (0.1 m^3^/h to 1 m^3^/h).

It is worth noting that treatment plants with a capacity greater than or equal to 10 m^3^/h are not expected to be constructed and operated. The investigated range covers the full range of typical hot-dip galvanizing industry capacity (e.g.**,** in Italy the highest capacity is nowadays 50,000 ton/year of steel processed, which would mean about 1 m^3^/h of waste solution to be treated; the Tecnozinco plant capacity is one order of magnitude lower).

### 3.2. Operating Costs

The yearly operating expenditure (OPEX) estimation was based on the value of the FCI, the cost of operating labor (COL), the cost of raw material (CRM), the cost of waste treatment (CWT) and the cost of utilities (CUT) following the indications of Turton et al. [21] (see Equation (37)).

(37) $$OPEX=0.18\times \mathrm{FCI}+2.73\times C_{OL}+1.23\times \left(C_{RM}+C_{WT}+C_{UT}\right)$$

The calculated value takes into account all the costs related to the total manufacturing costs: (i) Direct Costs, (ii) Fixed Manufacturing Costs and (iii) General Manufacturing Expenses. In this OPEX definition the depreciation is not taken into account: it is taken into account in the overall expenses (see following paragraph). Inputs for the calculation of the different OPEX items were collected and reported in Table 4.

C_RM_ concerns HCl make-up and reactants for the reactive crystallizer. The HCl industrial price is very variable since, in the relevant sector, it is a by-product of other processes and its price depends on several factors, such as the abundance in the specific geographical area or in the particular time period, and the incidence of transport costs. The chosen and reported price is mainly based on Tecnozinco knowhow. Moreover, the HCl makeup amount to consider is the difference between the design value (i.e.**,** the one resulting from the integration of the treatment chain in Tecnozinco’s plant) and the current Tecnozinco nominal consumption, as only this difference would result in an extra cost for the company. For the NH_4_OH and H_2_O_2_ reactants, Tecnozinco purchasing costs were considered as well. It is worth noting that all the selected purchasing costs are the highest in the relevant range reported in Table 4, thus conservatively assessing the worst scenario for the operating cost analysis.

C_WT_ is considered zero for the present process.

C_UT_ plant utilities consist of process water re-filling and electricity. Thermal energy is provided in the form of waste heat. Therefore, thermal energy cost is assumed to be zero. Electricity is mainly consumed by pumps, controls and switch cabinet cooling.

C_OL_, since the pilot plant principally operates automatically, it was considered only a quarter of an engineer position for maintenance and operation. With an assumed engineering salary of EUR 44,000/year this leads to EUR 11,000/year.

### 3.3. Profitability Analysis

For the yearly revenues (R) estimation, the following aspects have to be taken into account. Along with the direct revenues related to the selling or re-cycling of products (S_Product_), a saving for the reduced waste acid production and corresponding disposal (C_Disposal_), and for the enhanced production (*Enhanced Prod.*) for the optimal pickling performance of the baths have to be counted. In particular, S_Product_ refers to solid iron hydroxide, which is the direct saleable product, and the fluxing solution, which is the recyclable product. The latter is considered a revenue contribution as it is an avoided expense for the company.

In order to account for the avoided disposal cost (*C_Disposal_*), a saving flow rate was estimated as the flow rate corresponding at a Fe concentration disposal of 185 g/L as reported in Equation (38).

(38) $$W_{saving}=\frac{w_{Fe}^{WAS}\rho^{WAS}}{C_{Fe}^{Disposal}}$$

where $w_{Fe}^{WAS}$ is the mass flow rate of iron extracted from the pickling bath and thus processed in the treatment chain, $\rho^{WAS}$ is the waste acid solution density and the $C_{Fe}^{Disposal}$ is the highest mass concentration of iron at which the solution has to be disposed.

Another revenue contribution comes from increased production (*Added value of the Enhanced Prod.*). Pickling performances are dependent on several factors: temperature of the bath, acid and iron concentration, inhibitor choice and concentration [23]. A suitable combination of all these factors leads to the determination of optimal operating conditions for the pickling process. Under these conditions, the time required for the dissolution of the oxide layers covering the steel product is minimal and therefore the efficiency of the pickling bath is maximized.

**Figure 3:**
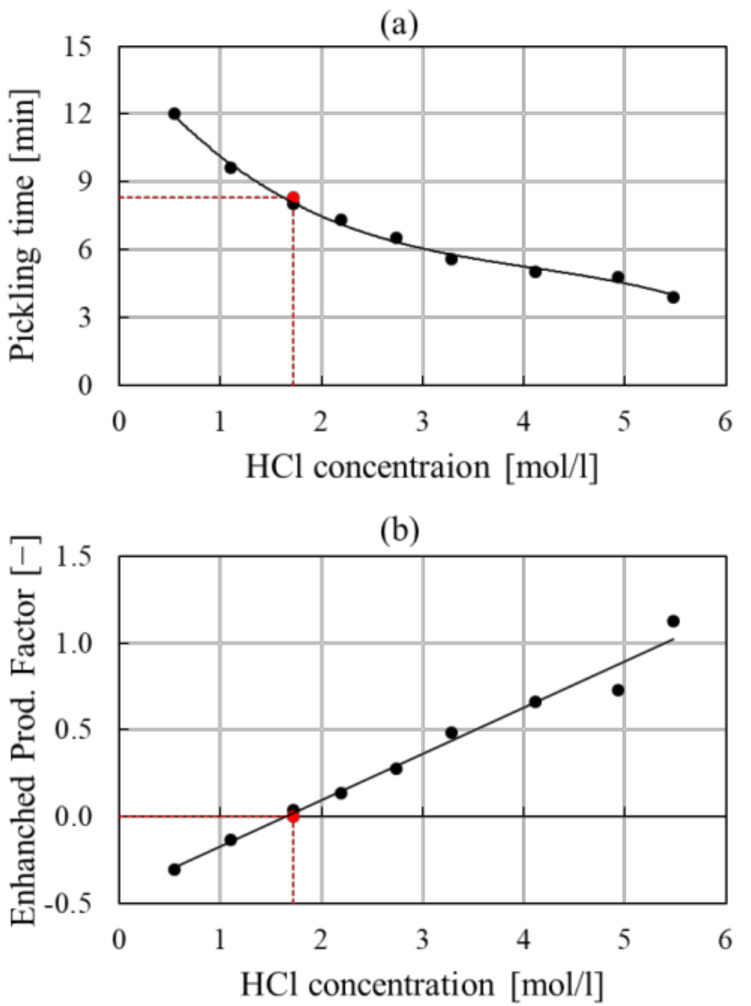
Minimum pickling time (**a**) and Enhanced Prod. Factor (**b**) vs. hydrochloric acid concentration. Red dot is the reference condition of the Tecnozinco pickling baths.

The implementation of the proposed process will affect mainly the composition of the bath in terms of acid and iron (II) concentration. In particular, literature values from Kleingarn were extrapolated and processed in order to derive a minimum pickling time curve with the hydrochloric acid concentration, reported in Figure 3a [24]. The minimum time is the pickling time corresponding to the optimal compositions of acid and iron. Figure 3b, on the contrary, shows the *Enhanced Prod. Factor* obtained by comparing a nominal operating condition of the Tecnozinco pickling baths, averaged over the data of one year of operation (HCl concentration: 1.7 mol/L; Fe (II) concentration: 137 g/L; average pickling time 8.3 min, red dot in Figure 3a) with data obtained in Figure 3a. This is a correction factor for the processed steel mass flow rate and expresses the variation of the pickling time, transformed into an increase or decrease in the processed material capacity of the plant, with the bath composition. Indeed, as the Tecnozinco nominal reference operating condition is almost an optimal condition of the Kleingran curve, for the reference concentration the *Enhanced Prod. Factor* does not show any variation in terms of productivity. However, if we move to a higher HCl operating concentration, increased production will result. Moreover, a reduction factor will be obtained if the acid working concentration is below the nominal case (refer to Figure 3b).

As defined, this is related to the particular plant operation, and it has to be tuned for the specific company considered. However, a typical composition for the relevant industrial sector could be used in order to generalize the *Enhanced Prod. Factor.*

Thus, the increased production *Enhanced Prod.* is expressed as follows:

(39) $$Enhanced\;Prod=Enhanced\;Prod.\;Factor\times w_{steel}$$

where $w_{steel}$ is the processed steel mass flow rate.

Finally, the yearly revenues were estimated as follows (Equation (40)):

(40) $$R=S_{Product}+C_{Disposal}+Added\;value\;of\;the\;Enhanced\;Prod.$$

where the “ Added value of the Enhanced Prod.” is the gain due to the *Enhanced Prod.* manufactured steel flow rate.

Table 5 summarizes the inputs used for the estimation of revenues together with the unitary cost for the selling or cost savings. As the iron (III) hydroxide market is very wide, the price depends on the particular application case: values vary from EUR 12/kg [25] for the wastewater treatment [26], to EUR 8.5/kg [27] for the painting industry, to EUR 0.6/kg as reported in “Xiamen Ditai Chemicals Co., Ltd.” webpage [28]. The Molbase Chemical E-commerce platform reports an average price of USD 17/kg based on more than 50 suppliers of the iron hydroxide product [29]. Thus, a value of EUR 2/kg was selected in order to be very conservative and to take also into account further possible treatment processes necessary to make it marketable.

The selected costs for the *Fluxing Solution* saving and the *Added value* of the *Enhanced Prod.* have been carefully discussed and agreed with the industrial partner, thus making analysis more reliable. Disposal costs faced by Tecnozinco S.r.l. include transportation to a far waste treatment plant in northern Italy (nearly 800 km). The disposal cost amounts to about EUR 80/ton, which is in agreement with values reported from Stocks et al. [30], while the additional average cost for transportation is around EUR 65/ton.

The simple straight-line depreciation method was applied by following the procedure described by Turton [18]: the total capital for depreciation was assumed equal to the fixed capital investment since a salvage value zero was taken as common custom for chemical plants and also to be more conservative. Note that the working capital was neglected, as the feed material is the industrial plant waste stream and the reactants employed in the process are already present on the industrial site.

The yearly plant expenses are evaluated as the sum of the annual manufacturing cost (OPEX) and the yearly depreciation. A fixed taxation rate of 33% of profits (corporate taxation rate in Italy), was assumed. Clearly, profit is the difference between annual revenues (R) and expenses. Thus, the net cash flow was evaluated as the net profit after tax plus the yearly depreciation. A discount rate equal to 4% was assumed.

All the inputs for the profitability analysis relevant to the Tecnozinco case study (ref. Future Size I in Table 2) are summarized in Table 6.

The CAPEX has been calculated according to the following equation (Equation (41)):

(41) $$CAPEX=FCI\;\frac{i\;{\left(1+i\right)}^{n}}{{\left(1+i\right)}^{n}-1}\;$$

**Figure 4:**
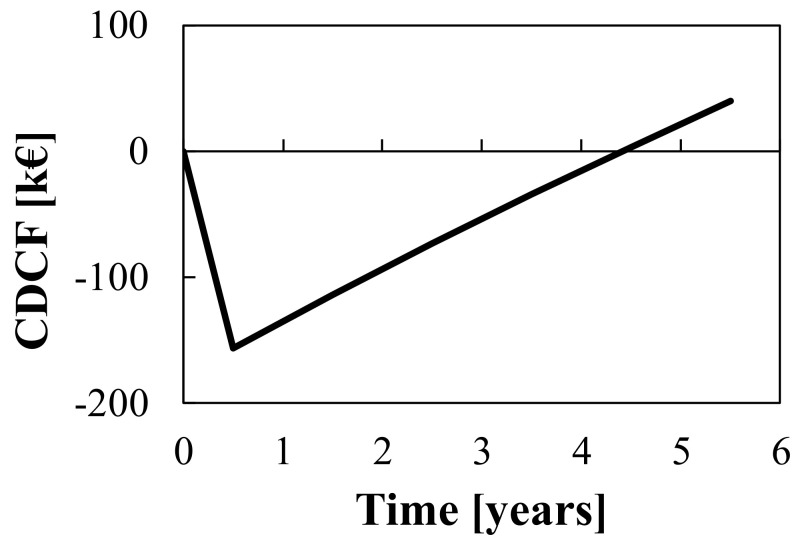
Cumulative discounted cash flow vs. time for the Tecnozinco case study.

The resulting cumulative discounted cash flow (CDCF) diagram, calculated as reported in Equations (42) and (43), is shown in Figure 4.

(42) $$CDCF_{k}=\overset{k}{\underset{z=1}{\sum }}DCF_{z}\;$$

(43) $$DCF_{k}=\frac{CF_{k}}{{\left(1+i\right)}^{k}}\;$$

Results are presented in terms of the main profitability indexes, whose definitions are reported in Table 7. As determined through calculation and also shown in Figure 4, a discounted payback period (DPBP) of 4 years is obtained. Thus, assuming a project duration of 5 years, the net present value (NPV) is about EUR 40,000, thus demonstrating the project’s profitability. The resulting discounted cash-flow rate of return (DCFROR) is 12.6%. Furthermore, it is worth noting that the economic analysis does not take into account the highly valuable benefit resulting from the reduction of the plant’s ecological burden.

## 4. Optimization Problem Formulation

Optimization analyses on the process performance in steady state operations were run on the gPROMS modelling platform. Optimization is required to create greater insight into the proposed process showing when integration into the traditional pickling industry could provide a significant benefit. The impact of key operating and design variables was investigated, and optimized configurations are presented in following section. As the Tecnozinco plant (ref. Future Size I in Table 2) is considered a small company in the relevant sector, a medium-size capacity optimized configuration is presented as well. Hot dip galvanizing plant capacity is characterized by the amount of steel processed over one year of operation; thus, this variable was considered as a scenario variable.

Moreover, the optimization analysis was aimed at identifying possible critical issues in the integration of the proposed process into the industrial site, as will be explained in the following paragraphs.

### 4.1. Optimization and Sensitivity Analysis with Operating Variables

**Figure 5:**
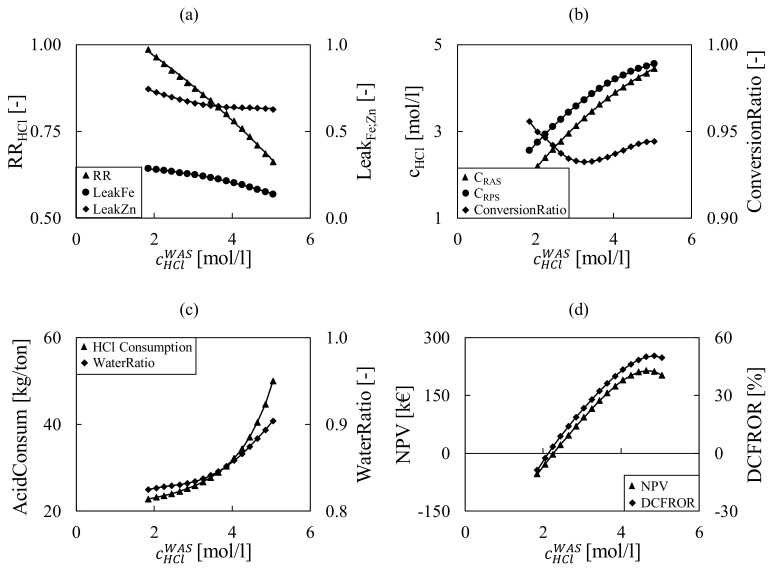
Sensitivity analysis with $c_{HCl}^{WAS}$ for the key variables in the DD (**a**), MD (**b**), Pickling and Integrated Process (**c**) and Economic (**d**) model sections.

As a preliminary example, a sensitivity analysis by varying the HCl concentration is reported in Figure 5. Profitability inputs reported in Table 6 were selected as the reference case. Steady state operations were studied, which impose the choice of the independent variable between the feed flow rate and the acid concentration of the waste stream, where the plant capacity is considered as a given data item in the model (scenario variable). For the optimization, the acid concentration was chosen as control variable in this study.

Undoubtedly, the HCl concentration in the pickling bath, and consequently the Fe concentration as predicted from the Kleingarn curve [24], strongly affects the flow rates, composition and performance parameters of the integrated system. Figure 5 shows the main operating variables in the different sections of the integrated model, namely DD, MD, pickling and integrated process, by allowing the HCl waste acid concentration to range from 2 to 5 mol/L. As shown in Figure 5a, declining performances are detected in the DD recovery ratio (RR) (defined in Equation (11)), as lower recovery values are observed when inlet acid concentration increases. Moreover, although lower iron concentrations correspond to higher acid concentrations according to the Kleingran curve, the benefit in reduced metals leakage $Leak_{Fe;Zn}\;($defined in Equation (12)) is not so prominent. *ConversionRatio* in the MD, defined in Equation (20) as the ratio between the amount of acid recovered and the MD inlet acid amount, presents a non-monotonic trend. At low $c_{HCl}^{WAS}$ concentrations the *ConversionRatio* decreases despite the increasing of acid concentration in the recovered pickling solution, as the recovered amount is lower compared to the inlet acid (in the recovered acid solution). Then, at the higher waste acid concentrations, which means higher acid concentrations in the recovered acid solution, the water vapor pressure decreases, thus reducing the water extraction through the MD unit, causing an increase of the conversion ratio (Figure 5b). Performances of the integrated process are expressed with the acid consumption *AcidCosum* (Equation (6)), i.e.**,** the make-up acid for processed steel, and the *WaterRatio* (Equation (33)), which is significant of the plant water demand; both of these decline rapidly as the HCl pickling bath concentration increases (Figure 5c). The profitability analysis, expressed by the NPV (defined in Equation (44)), which is a standard method for using the time value of money to appraise long term project (in the particular case 5 years project as specified in Table 6), and DCFROR (defined in Equation (45)) indexes in Figure 5d), predicts a maximum for an acid concentration of around 4.5 mol/L. However, at this maximum profitability the consumption of water and acid would be significantly higher than the galvanizing companies’ standard average consumption.

Based on these sensitivity analysis results, an optimized configuration is gained through the gPROMS process optimization tool simulation. Inputs for the optimization problem together with results configuration are presented in Table 8. In particular, the Net Present Value has been selected as objective function to be maximized, while using the waste acid feed flow rate and composition as control variables, for which the “optimizing” set of values was identified. The fixed reference scenario of a plant steel treatment capacity of 2030 kg/h has been set. Finally, equality and inequality constraints have been imposed in order to fix the acid consumption and limit the variation of control variables within realistic ranges, as reported in Table 8.

The optimum value, for the Tecnozinco plant (ref. Future Size I in Table 2), is not so far from the original values of the design variables since the process was designed by choosing the best operating conditions after performing several sensitivity analyses [3] (see Table 6 as benchmark). In addition, the NPV for the optimized configuration is constrained by the acid consumption, which was chosen to be comparable to the average acid consumption reported by galvanizing companies. In fact, it results a limitation for the acid concentration and, consequently, for the net profit (see Table 8). The corresponding DPBP is 3.6 years.

### 4.2. Optimization with Operating and Design Variables

Profitability of the process strongly depends on the design variables as the capital cost of investment increases with the areas of the membrane units. In Table 9, the result of an optimization with both the operating and the design variable is proposed, with the same purpose of maximizing the NPV. Optimized values are comparable with results obtained for the operating variables optimization configuration (see Table 8): feed flow and acid concentration are quite similar, while the different membrane areas determine the variation in the resulting net profit.

A scaled-up scenario with a processed steel capacity of 10,000 kg/h (ref. Future Size II in Table 2) was also analyzed as presented in Table 9. Of course, economy of scale guarantees a higher economic return.

Through up-scaling, over EUR 913,000 of NPV is reachable and the DPB decreases from 3.4 to 2.2 years.

#### Trade-Off Solution between Profitability and Environmental Issue

The profitability aspect was considered so far. A strong limitation was imposed by fixing the acid consumption of the plant, which bounds the acid make-up demand. A further critical issue related to the integration of the process in the industrial plant is explored in the present section. The process was proposed in order to avoid exhausted process fluids, and this beneficial aspect was taken into account by considering the money saved by avoiding disposal.

Another crucial aspect concerns the reduction of water use and wastewater production. The DD unit requires a drawing solution flow rate comparable to the waste acid flow rate. Almost 90% of the drawing solution is taken from process water. In order to reduce the freshwater consumption, a threshold value has to be considered to obtain the highest profit along with a low water consumption.

The trade-off value was evaluated using a multi-objective optimization approach, in order to find out the preferred solutions that maximize the NPV objective function while minimizing the *WaterRatio* variable, defined in Equation (33) as the ratio between the fresh process water demand and the drawing DD feed solution. To this purpose, a Pareto Frontier was found by using the ε-constraints method [31], constructed by using the maximum NPV for a given *WaterRatio* value, thus converting a multi-objective analysis to a parametrized single-objective one.

**Figure 6:**
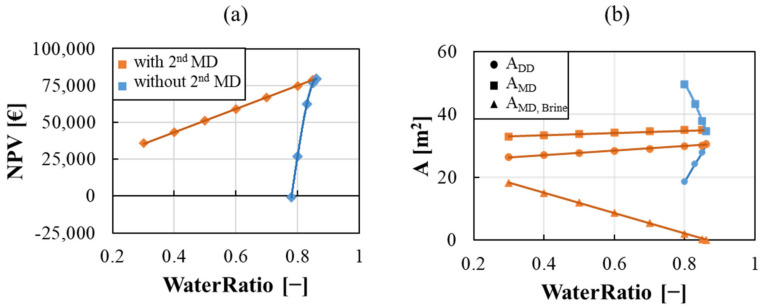
Pareto frontier (**a**) and optimal value of the total membrane area (**b**) vs. *WaterRatio* for the scenario with (orange) and without (blue) the MD unit for outlet brine treatment.

In Figure 6, results for the Tecnozinco case study are reported by evaluating the NPV with the variation of the *WaterRatio* up the value of 90%. A strong sensitivity is observed, as a slight reduction of the water utilization leads to a significant increase of the MD membrane area and thus of the costs. As a consequence, the NPV of the plant is firmly reduced, making this configuration unprofitable for a *WaterRatio* of about 80% as shown in Figure 6a (blue line).

**Figure 7:**
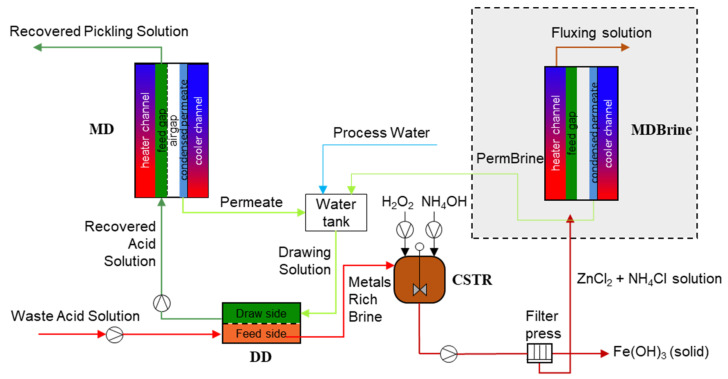
Schematic representation of the main units of the integrated process with additional MD brine unit.

In the same figure, a second scenario is also shown. In order to reduce the water demand, a second MD Brine unit can be included in the process in order to concentrate the fluxing solution and recover a part of the process water to be used as drawing media in the diffusion dialysis (representation of this second configuration is shown in Figure 7).

## 5. Conclusions

The aim of the present work was to demonstrate the economic feasibility of a hybrid process, where two membrane technologies are coupled with a reactive precipitation unit in order to valorize the waste liquor of hot-dip galvanizing companies. A detailed economic analysis has been presented by evaluating the FCI of the treatment process with a capacity ranging from 0.1 m^3^∙h**^−^**^1^ to 10 m^3^∙h**^−^**^1^, thus covering all typical hot-dip galvanizing industry capacities.

The gPROMS simulation platform was employed (i) to simulate steady state operations of the process and (ii) to perform optimization analyses. The optimization problem formulation was aimed to maximize the net present value of the process using, firstly, operating parameter (acid concentration) and then, also the design variables (membrane modules areas) as control variables. The process was found profitable for the low size capacity of the Tecnozinco plant and, more importantly, the profitability is much more evident at larger scales. Indeed, for the real industrial scenario (Tecnozinco, Carini, Italy) plant, results show a DPBP of 4 years and, assuming a project duration of 5 years, a NPV value of about EUR 40,000 was obtained. Furthermore, the reduction of ecological burden gives a significant valuable benefit in all the scenarios investigated.

The above findings indicate that the technology is profitable and can be easily integrated into industrial plants. The multi-objective optimization highlighted the possibility of increasing the NPV up to about EUR 75,000, with a water ratio of 85%, by including in the integrated process a second MD unit, which allows the reduction of water use. The proposed technology shows a huge potential for the relevant industry sector as it provides a solution for the simultaneous reduction of costs and environmental impact, thus moving the pickling process towards a higher level of sustainability. Future research directions are oriented towards the further testing of the system at the pilot scale, followed by the system scale-up and application to real scenarios.

## Figures and Tables

**Table 1:** gPROMS main model equations for the different sections of the integrated process. Density in Equation (31) is estimated according to [17].

Pickling		
$Cons/Prod_{i,p}=\frac{{ʋ}_{i,p}}{{ʋ}_{HCl,p}}Cons/Prod_{HCl,p}\times \frac{MW_{i}}{MW_{HCl}}$, Ɐ *i* ≠ HCl		(1)
$Cons/Prod_{HCl,p}=-k_{HCl}\times \chi_{p}\times w_{steel}$		(2)
$w_{j}^{RPS}+w_{j}^{MU}+w_{j}^{entr_{in}}+k_{j}\times w_{steel}=w_{j}^{WAS}+w_{j}^{entr_{out}}+w_{j}^{evap}$		(3)
$\;F^{WAS}\times c_{FeCl_{2}}^{WAS}\times MW_{FeCl_{2}}=k_{FeCl_{2}}\times w_{steel}+w_{FeCl_{2}}^{RPS}+w_{FeCl_{2}}^{entr,in}$		(4)
$c_{FeCl_{2}}^{WAS}=-0.544\times c_{HCl}^{WAS}+3.581$		(5)
$HCl_{comsumption}=\frac{F^{MU}\cdot \rho^{MU}}{w_{steel}}$		(6)
*p* pickling reactions	Fe_2_O_3_ + Fe + 6HCl = 3FeCl_2_ + 3H_2_O Fe_3_O_4_ + Fe + 8HCl = 4FeCl_2_ + 4H_2_O	
*i* pickling components	Fe_2_O_3_, Fe_3_O_4_, Fe, FeCl_2_, HCl, ZnCl_2_, H_2_O	
*j* components	HCl, FeCl_2_, ZnCl_2_, H_2_O	
Diffusion Dialysis		
$J_{j}^{DD}=\underset{m}{\sum }\left(P_{j,m}\frac{c_{m}^{WAS}-c_{m}^{RAS}+c_{m}^{MRB}-c_{m}^{DS}}{2}\;\right)$	Ɐ *j* ≠ H_2_O *m =* HCl, FeCl_2_, ZnCl_2_	(7)
$J_{H_{2}O}=P_{H_{2}O}\times \Delta \Pi +\underset{m}{\sum }\beta_{m}J_{j}^{DD}$		(8)
$w_{j}^{MRB}=w_{j}^{WAS}-J_{j}^{DD}\times MW_{j}\times A_{DD}^{tot}$	Ɐ *j* ≠ H_2_O	(9)
$w_{j}^{MRB}+w_{j}^{RAS}=w_{j}^{WAS}+w_{j}^{DS}$		(10)
$RR_{HCl}=\frac{w_{HCl}^{RAS}-w_{HCl}^{DS}}{w_{HCl}^{WAS}}$		(11)
$Leak_{Fe;Zn}=\frac{w_{Fe;Zn}^{RAS}-w_{Fe;Zn}^{DS}}{w_{Fe;Zn}^{WAS}}$		(12)
$FlowRatio=\frac{F^{DS}}{F^{WAS}}$		(13)
$n_{AEM}=\frac{A_{DD}^{tot}}{L\times W}$		(14)
Membrane Distillation		
$J_{j}^{MD}=a_{j}{\left(\frac{F^{RAS}\cdot \rho^{RAS}}{n_{feed}}\;\right)}^{2}-b_{j}\left(\frac{F^{RAS}\cdot \rho^{RAS}}{n_{feed}}\;\right)+c_{j}$	$a_{j}$ $,\;b_{j}$ $,\;c_{j}$ $=f(c_{HCl}^{RAS})$	(15)
$J_{FeCl_{2}}^{MD}=J_{ZnCl_{2}}^{MD}=0$		(16)
$w_{j}^{Perm}=A_{MD}\times J_{j}^{MD}\times n_{feed}$		(17)
$w_{j}^{RPS}=w_{j}^{RAS}-w_{j}^{Perm}$		(18)
$A_{MD}^{tot}=A_{MD}\times n_{feed}$		(19)
$ConversionRatio=\frac{w_{HCl}^{RPS}}{w_{HCl}^{RAS}}$		(20)
Reactive Crystallizer		
$\varsigma_{r_{1}}={ʋ}_{HCl}^{r_{1}}\left(c_{HCl}^{MRB}-10^{-pH}\right)F^{MRB}$		(21)
$\varsigma_{r_{2}}={ʋ}_{FeCl_{2}}^{r_{2}}\times c_{FeCl_{2}}^{MRB}\times F^{MRB}$		(22)
$w_{NH_{4}OH}^{base}=-\left(\frac{{ʋ}_{NH_{4}OH}^{r_{1}}}{{ʋ}_{HCl}^{r_{1}}}\varsigma_{r_{1}}+\frac{{ʋ}_{NH_{4}OH}^{r_{2}}}{{ʋ}_{FeCl_{2}}^{r_{2}}}\varsigma_{r_{2}}\right)MW_{NH_{4}OH}\times f_{NH_{4}OH}^{excess}$ $w_{H_{2}O_{2}}^{oxidant}=-\frac{{ʋ}_{H_{2}O_{2}}^{r_{2}}}{{ʋ}_{FeCl_{2}}^{r_{2}}}\varsigma_{r_{2}}MW_{H_{2}O_{2}}\times f_{H_{2}O_{2}}^{excess}$		(23)
		(24)
$\left\{\begin{array}{c} w_{NH_{4}OH}^{fluxing}=\left(1-\frac{1}{f_{NH_{4}OH}^{excess}}\right)w_{NH_{4}OH}^{base}\left(1-\alpha \right)\\ w_{H_{2}O_{2}}^{fluxing}=\left(1-\frac{1}{f_{H_{2}O_{2}}^{excess}}\right)w_{H_{2}O_{2}}^{oxidant}\left(1-\alpha \right)\\ w_{NH_{4}Cl}^{fluxing}=\left(\frac{{ʋ}_{NH_{4}Cl}^{r_{1}}}{{ʋ}_{HCl}^{r_{1}}}\zeta_{r_{1}}+\frac{{ʋ}_{NH_{2}Cl}^{r_{2}}}{{ʋ}_{Fell_{2}}^{r_{2}}}\zeta_{r_{2}}\right)MW_{NH_{4}Cl}\left(1-\alpha \right)\\ w_{j}^{fluxing}=\left(w_{j}^{\mathrm{MRB}}+\left(\frac{{ʋ}_{j}^{r_{1}}}{{ʋ}_{HCl}^{r_{1}}}\zeta_{r_{1}}+\frac{{ʋ}_{j}^{r_{2}}}{{ʋ}_{FeCl_{2}}^{r_{2}}}\zeta_{r_{2}}\right)MW_{j}\right)\left(1-\alpha \right) \end{array}\right.$		(25)
		(26)
		(27)
		(28)
$\{\begin{array}{c} w_{n}^{slurry}=w_{n}^{fluxing}\times \frac{\alpha }{1-\alpha }\;\;\;\;\;\left(liq\right)\\ w_{Fe{\left(OH\right)}_{3}}^{slurry}={ʋ}_{Fe{\left(OH\right)}_{3}}^{r_{2}}c_{FeCl_{2}}^{MRB}F^{MRB}\times MW_{Fe{\left(OH\right)}_{3}} \end{array}$		(29)
		(30)
*r* reactions	$r_{1}$: HCl + NH_4_OH = NH_4_Cl + H_2_O $r_{2}$: FeCl_2_ + 1/2 H_2_O_2_ + 2NH_4_OH = Fe(OH)_3(s)_ + 2NH_4_Cl	
*n* reactor components	*n* = *j* + NH_4_OH, H_2_O_2_, NH_4_Cl	
*base:*	NH_4_OH, H_2_O	
*oxidant:*	H_2_O_2_, H_2_O	
Density		
$\frac{1}{\rho }=v_{mix}=x_{w,H_{2}O}\cdot v_{H_{2}O}+\underset{i}{\sum }v_{i}=x_{w,H_{2}O}\times v_{H_{2}O}+\underset{i}{\sum }x_{w,i}v_{app,i}$		(31)
Integrated Process		
*Connectivity equations*		
$w_{j}^{DS}=w_{j}^{Perm}+w_{j}^{PW}$		(32)
$WaterRatio=\frac{F^{PW}}{F^{DS}}$		(33)

**Table 2:** FCI analysis at different treatment capacity.

	Treatment Capacity (Feed Flow Rate)			
Cost Items [€]	Pilot Size 0.02 m^3^ h^−1^	Future Size I 0.1 m^3^ h^−1^	Future Up-Scaled Size II 1 m^3^ h^−1^	Future Up-Scaled Size III 10 m^3^ h^−1^
Mechanical	6300	6300	7600	16,000
Hydraulic	8600	16,500	33,000	82,500
Actuators	4200	5400	6500	27,000
Sensors	13,900	12,500	12,500	12,500
Electrical	12,600	16,000	16,000	16,000
Total Material Costs (excl. Modules)	45,600	56,700	75,600	154,000
MD Module	10,000	13,500	36,000	84,000
DD Module	8000	9800	58,400	418,000
Membrane Module cost	18,000	23,300	94,400	502,000
Total Material Costs (incl. Modules)	63,600	80,000	170,000	656,000
Freight, insurance, taxes	2000	2000	4000	6000
Logistics, Ordering, Desk	5200 (1.4 × PM) ^1^	5200 (1.4 × PM) ^1^	6200 (1.7 × PM) ^1^	12,600 (3.4 × PM) ^1^
Documentation	2600 (0.7 × PM) ^1^	2600 (0.7 × PM) ^1^	3000 (0.8 × PM) ^1^	6200 (1.7 × PM) ^1^
Assembly	24,000 (8.3 × PM) ^2^	24,000 (8.3 × PM) ^2^	48,000 (8.3 × PM) × 2 ^2^	240,000 (8.3 × PM) × 10 ^2^
Commissioning and Training	8100 (1.4 × PM) ^3^	8100 (1.4 × PM) ^3^	9900 (1.7 × PM) ^3^	19,600 (3.4 × PM) ^3^
Development	8100 (1.4 × PM) ^3^	8100 (1.4 × PM) ^3^	9900 (1.7 × PM) ^3^	19,600 (3.4 × PM) ^3^
**Subtotal**	113,600	128,000	251,000	960,000
Technology Provider Fee (=25% of subtotal)	28,400	32,000	62,700	240,000
**FCI (total)**	**142,000**	**160,000**	**313,700**	**1,200,000**
$Lump\;Factor=\frac{FCI}{Membrane\;Modules\;Cost}$	*7.9*	6.9	3.3	2.4

^1^ Engineer 2 ^2^ 8 Engineer + 5.5 Assembler ^3^ Senior Engineer.

**Table 3:** Estimated salaries [19] used for cost items determination in Table 2.

Position	Salary
Engineer	EUR 44,000/year
Senior Engineer	EUR 70,000/year
Assembler	EUR 30,000/year

**Table 4:** Unitary costs for OPEX estimation.

OPEX Items	Cost Position	Unitary Cost ^1^	Selected Values
Raw Material (inputs for C_RM_ calc.)	HCl Make-Up	30–125 €/ton	125 €/ton
	Alkaline reactant	0.1–0.55 €/L	0.55 €/L
	Oxidizing reactant	0.135–0.38 €/kg	0.38 €/kg
Waste treatment (inputs for C_WT_ calc.)	-	-	-
Utilities (inputs for C_UT_ calc.)	Process Water	-	0.95 €/m^3^
	Electricity	-	0.2 €/kWh
Operating Labor C_OL_		-	11,000 €/year

^1^ Unitary cost have been derived from the website Echemi [22].

**Table 5:** Unitary costs for Revenues calculation.

Revenues Inputs	Unitary Cost	Selected Values
Iron (III) hydroxide	EUR 0.6–14/kg ^1^	EUR 2/kg
Fluxing solution	EUR 0.06/kg	EUR 0.06/kg
Waste acid disposal saving	EUR 40–160/ton ^2^	EUR 145/ton
Added value of the Enhanced Prod.	EUR 0.045/kg	EUR 0.045/kg

^1^ The range was estimated according to [25,27,28,29] ^2^ The range was estimated according to [1,30].

**Table 6:** Profitability analysis inputs for the Tecnozinco case study (ref. Future Size I in Table 2).

Profitability Analysis Inputs		Unit
Feed flow rate (F_WAS_)	130	L/h
Process Steel	2030	kg/h
DD total area (A_DD_)	25	m^2^
MD total area (A_MD_)	43.5	m^2^
FCI	160	EUR k
**CAPEX**	36	EUR k/year
OPEX	125	EUR k/year
R	180	EUR k/year
Project duration (n)	5	y
Time for plant construction	0.5	y

**Table 7:** Profitability key parameters.

Economics	
$NPV=\overset{n}{\underset{k=1}{\sum }}DCF_{k}$	(44)
$\overset{n}{\underset{k=1}{\sum }}\frac{CF_{k}}{{\left(1+DCFROR\right)}^{k}}=0$	(45)
PBP= time to recover the FCI after start-up	(46)

**Table 8:** Optimization problem and results for the scenario with operating variables as control variables.

Optimization Problem				Results
Fixed reference condition (given scenario)		$\mathrm{Plant}\;\mathrm{throughput}\;(w_{steel}$)		2030 kg/h
Control variables		$\mathrm{Feed}\;\mathrm{flow}\;(F^{WAS}$) or $\mathrm{composition}\;(c_{HCl}^{WAS})$		134 L/h
				2.7 mol/L
Objective function (to be maximised)		NPV		EUR 63,777
Constraints	Equality	Steady state operation		
		Acid consumption	25 kg/ton *	25 kg/ton
	Inequality	Channel velocity	0.1 cm/s < v_DD_ & v_MD_ < 3 cm/s	v_DD_ 0.88 cm/s v_MD_ 0.17 cm/s
		FlowRatio	$0.5\;<\;\frac{F^{DS}}{F^{WAS}}$ < 1.5	1.09

* Acid consumption data provided by AIZ (The Italian Galvanizing Association) [1].

**Table 9:** Optimization problem and results for the scenario with operating + design variables as control variables.

Optimization Problem				Results	
Fixed reference condition (given scenario)		Plant throughput ($w_{steel}$)		2030 kg/h	10,000 kg/h
Control variables		Feed flow ($F^{WAS}$)		142 L/h	842 L/h
		Composition ($c_{HCl}^{WAS})$		2.86 mol/L	3.22 mol/L
		$\mathrm{DD}\;\mathrm{membrane}\;\mathrm{area}\;(A_{DD}$)		30.6 m^2^	231 m^2^
		$\mathrm{MD}\;\mathrm{membrane}\;\mathrm{area}\;(A_{MD}$)		34.7 m^2^	217 m^2^
Objective function (to be maximised)		NPV		EUR 79,631	EUR 913,000
Constraints	Equality	Steady state operation			
		Acid consumption	25 kg/ton *	25 kg/ton	25 kg/ton
	Inequality	Channel velocity	0.1cm/s < v_DD_	v_DD_ 0.76 cm/s	v_DD_ 0.6 cm/s
			v_MD_ < 3cm/s	v_MD_ 0.22 cm/s	v_MD_ 0.22 cm/s
		FlowRatio	$0.5\;<\;\frac{F^{DS}}{F^{WAS}}$ < 1.5	1.07	1.05

* Acid consumption data provided by AIZ (The Italian Galvanizing Association) [1].

## Acronyms

AEM		Anionic Exchange Membrane
AIZ		Italian Galvanizing Association
BAT		Best Available Techniques
C		Cost
CAPEX		Capital Expenditures
CDCF		Cumulative Discounted Cash Flow
CF		Cash Flow
DCF		Discounted Cash Flow
DCFROR		Discounted Cash Flow Rate of Return
DPBP		Discounted Payback Period
CSTR		Continuous Stirred Tank Reactor
DCMD		Direct Contact Membrane Distillation
DD		Diffusion Dialysis
DPBP		Discounted Payback Period
E		Expenses
EGGA		European General Galvanizers Association
FCI		Fixed Capital Investment
IPPC		Integrated Pollution Prevention and Control
MD		Membrane Distillation
N		Equipment Cost Attribute
NPV		Net Present Value
OPEX		Operating Expenditures
R		Revenues
S		Selling
**Nomenclature**		
A	$[m^{2}$]	area
c	$\left[\frac{mol}{l}\right]$	molar concentration
Cons/Prod	$\left[\frac{kg}{h}\right]$	consumption/production
F	$\left[\frac{l}{s}\right]$	volumetric flow rate
*f*	[-]	multiplicative factor
*i*	[%]	discount rate
J	$\left[\frac{mol}{m^{2}\cdot s}\right]$	molar flux
k	$\left[\frac{kg}{kg_{steel}}\right]$	kinetic constant
L	[m]	length
*n*	[y]	project duration
$n_{AEM}$	[-]	DD membranes number
$n_{feed}$	[-]	MD feed number
M *W*	$\left[\frac{g}{mol}\right]$	molar mass
P	$\left[\frac{m}{s}\right]$	diffusive permeability
RR	[%]	recovery ratio
W	[m]	width
w	$\left[\frac{kg}{h}\right]$	mass flow rate of the integrated process streams
α	[-]	amount of solution trapped in the humid cake
β	[-]	hydration number
ς	[-]	difference of value
ζ	[-]	extent of reaction
π	[bar]	osmotic pressure
ρ	$\left[\frac{g}{l}\right]$	density
υ	[-]	stoichiometric coefficient
χ	[-]	conversion
**Subscripts and Superscripts**		
DD		diffusion dialysis
DS		draw solution
entr, in		entrainment stream, inlet
entr, out		entrainment stream, outlet
evap		evaporating stream
*galv.steel*		galvanized steel
*i*		pickling component *i* i.e., Fe_2_O_3_, Fe_3_O_4_, Fe, FeCl_2_, HCl, ZnCl_2_, H_2_O
*j*		component *j*, i.e., HCl, FeCl_2,_ ZnCl_2_, H_2_O
*k*		generic year
*m*		component *m* i.e., HCl, FeCl_2,_ ZnCl_2_
MD		membrane distillation
MRB		metals-rich brine
MS		metal sludge
MU		make-up
*n*		reactor components *n,* i.e., HCl, FeCl_2,_ ZnCl_2_, H_2_O NH_4_OH, H_2_O_2_, NH_4_Cl
*p*		pickling reactions
*perm*		permeate
*PW*		process water
*r*		reactions
RAS		recovered acid solution
RPS		recovered pickling solution
*s*		pickling streams
*tot*		total
*u*		membrane unit
WAS		waste acid solution in the integrated process
